# Genomic and evolutionary features of two AHPND positive *Vibrio parahaemolyticus* strains isolated from shrimp (*Penaeus monodon*) of south-west Bangladesh

**DOI:** 10.1186/s12866-019-1655-8

**Published:** 2019-12-03

**Authors:** Shawon Ahmmed, Md. Abdullah-Al-Kamran Khan, Md. Mostavi Enan Eshik, Nusrat Jahan Punom, Abul Bashar Mir Md. Khademul Islam, Mohammad Shamsur Rahman

**Affiliations:** 1 Aquatic Animal Health Group, Department of Fisheries, University of Dhaka, Dhaka, 1000 Bangladesh; 2 Department of Genetic Engineering and Biotechnology, University of Dhaka, Dhaka, 1000 Bangladesh

**Keywords:** *Vibrio parahaemolyticus*, Acute hepatopancreatic necrosis disease, AHPND, Genome sequencing, Virulence, *pir*A, *pir*B, Phylogenetics

## Abstract

**Background:**

Due to its rapid lethal effect in the early development stage of shrimp, acute hepatopancreatic necrosis disease (AHPND) has been causing great economic losses, since its first outbreak in southeast China in 2009. *Vibrio parahaemolyticus*, carrying the *pir*A and *pir*B toxin genes is known to cause AHPND in shrimp. The overall objective of this study was to sequence the whole genome of AHPND positive *V. parahaemolyticus* strains isolated from shrimp (*Peneaus monodon*) of the south-west region of Bangladesh in 2016 and 2017 and characterize the genomic features and emergence pattern of this marine pathogen.

**Results:**

Two targeted AHPND positive *V. parahaemolyticus* strains were confirmed using PCR with 16S rRNA, *ldh*, AP3 and AP4 primers. The assembled genomes of strain MSR16 and MSR17 were comprised of a total of 5,393,740 bp and 5,241,592 bp, respectively. From annotation, several virulence genes involved in chemotaxis and motility, EPS type II secretion system, Type III secretion system-1 (T3SS-1) and its secreted effectors, thermolabile hemolysin were found in both strains. Importantly, the ~ 69 kb plasmid was identified in both MSR16 and MSR17 strains containing the two toxin genes *pir*A and *pir*B. Antibiotic resistance genes were predicted against β-lactam, fluoroquinolone, tetracycline and macrolide groups in both MSR16 and MSR17 strains.

**Conclusions:**

The findings of this research may facilitate the tracking of pathogenic and/or antibiotic-resistant *V. parahaemolyticus* isolates between production sites, and the identification of candidate strains for the production of vaccines as an aid to control of this devastating disease. Also, the emergence pattern of this pathogen can be highlighted to determine the characteristic differences of other strains found all over the world.

## Background

Asian shrimp farming industry has encountered enormous production losses because of a *Vibrio* caused disease, known as the early mortality syndrome/acute hepatopancreatic necrosis disease (EMS/AHPND) [1]. AHPND is a shrimp bacterial disease which causes high mortality of cultivated penaeid shrimps commonly occur within the first 30 days after stocking in grow-out ponds [2]. Since 2009, AHPND was first recorded in shrimp farms of southern China [3], in 2010 in Island of Hainan [2], in Vietnam and Malaysia in 2011 [4] and subsequently it spread in the eastern part and other culture areas of Thailand in 2012 [4]. Worldwide the production loss of shrimp farming due to AHPND was estimated at about more than $1 billion per year [5]. In Bangladesh, AHPND positive *Vibrio parahaemolyticus* were first reported in 2017 [6].

The AHPND affected shrimp shows a pale and atrophied hepatopancreas along with an empty stomach and midgut [3]. The moribund shrimps usually harbor some pathological features like- enlarged hepatopancreatic nuclei, sloughed HP cells-blister-like (B), fibrilla (F), resorptive (R) cells, and the diseased shrimps frequently suffer from secondary bacterial infections [3]. The causative agent of AHPND in shrimp is *Vibrio parahaemolyticus*; a gram-negative rod-shaped bacterium mainly inhabitant in warm marine and estuarine environment, and distributes throughout the world [3, 7]. AHPND causing *V. parahaemolyticus* possesses ~ 69 kb plasmid encoding toxin genes *pir*A and *pir*B [3, 8] which are similar to Photorhabdus insect-related (*pir*) toxin [9] which is one of the major causal factors reported. Moreover, two sets of the type III secretion system (T3SS1 and T3SS2) possessed by *V. parahaemolyticus* are also considered as an important virulence factor of this organism [10]. Though all strains of *V. parahaemolyticus* contain T3SS1, only the human clinical strains possess T3SS2 [10]. AHPND positive *V. parahaemolyticus* strains do not contain TDH, TRH, and T3SS2 which are known virulence factors affecting humans [11]. Amplification of species-specific gene *ldh* (lecithin dependent hemolysin) [12] is utilized to detect *V. parahaemolyticus* isolates whereas AP3 [13] and AP4 [14] primers are commonly used to identify the AHPND positive strains.

Nowadays, whole genome sequencing (WGS) has become a popular tool for the identification and detection of bacterial outbreaks in aquaculture [15]. In whole genome sequencing, all of the single nucleotide polymorphisms (SNPs) are used to confirm the epidemiological links of outbreak strains with higher typing resolution [16]. In this study, we have sequenced two AHPND positive *V. parahaemolyticus* strains (MSR16 and MSR17) which were isolated from shrimp farms of the south-west region of Bangladesh and this is the very first genome sequencing report of AHPND positive *V. parahaemolyticus* strains isolated from shrimps of Bangladesh. Subsequently, we analyzed their genomic features associated with virulence and other factors. Finally, we have performed phylogenetic analyses using several genomic features of this bacteria to find out the relations between the outbreak causing strains around the globe with our sequenced strains.

## Results

### Identification of the AHPND positive strains

**Fig. 1:**
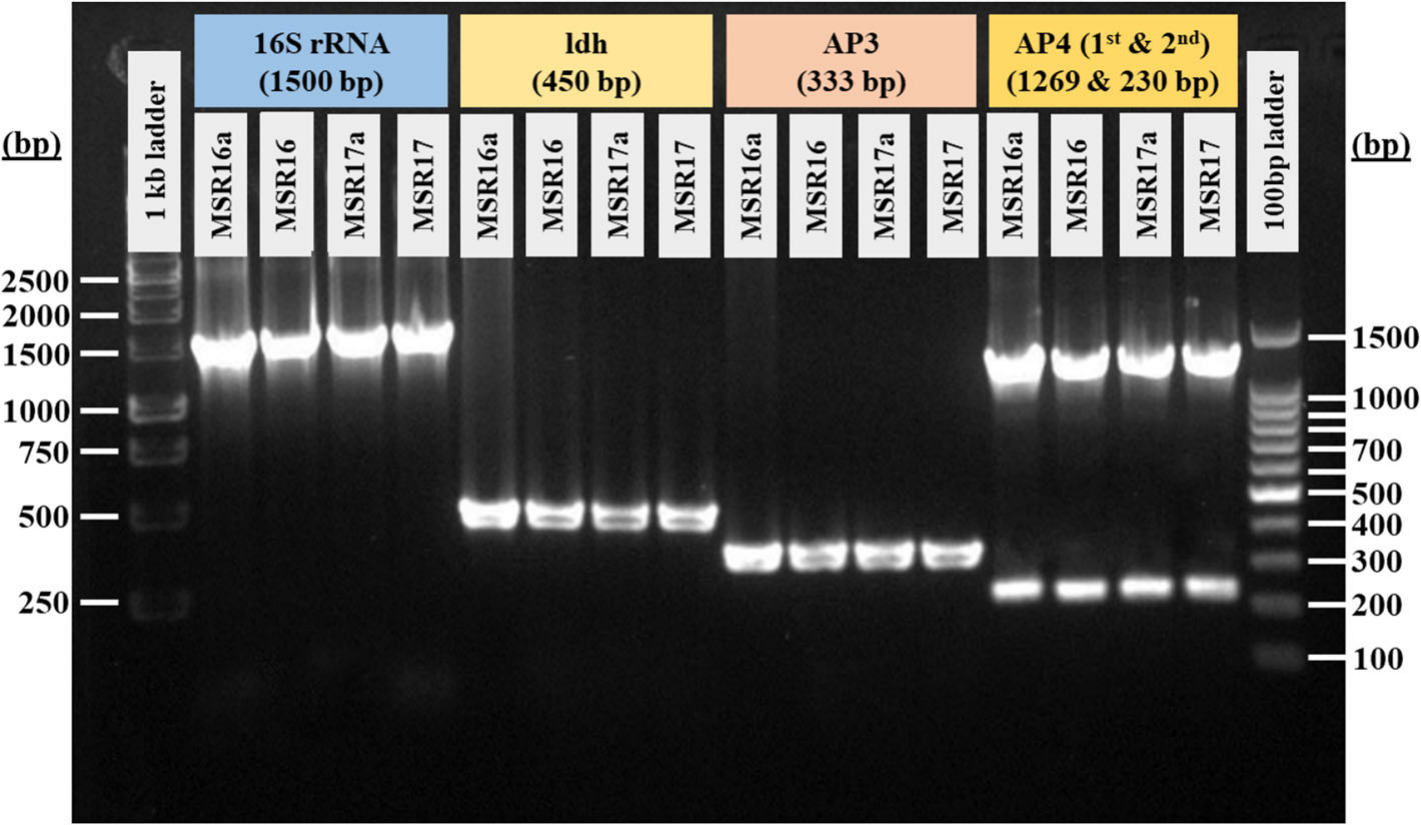
Molecular identification of the AHPND positive *V. parahaemolyticus* strain MSR16 and MSR17. (MSR16a and MSR17a are replicates of MSR16 and MSR17, respectively)

Molecular identification and characterization of suspected AHPND positive *V. parahaemolyticus* isolates were done using 16S rRNA, *ldh*, AP3 and AP4 primers PCR (Fig. 1). MSR16 (isolated in 2016) and MSR17 (isolated in 2017) strains were finally sequenced for whole genome sequencing.

### Features of the assembled genomes

**Fig. 2:**
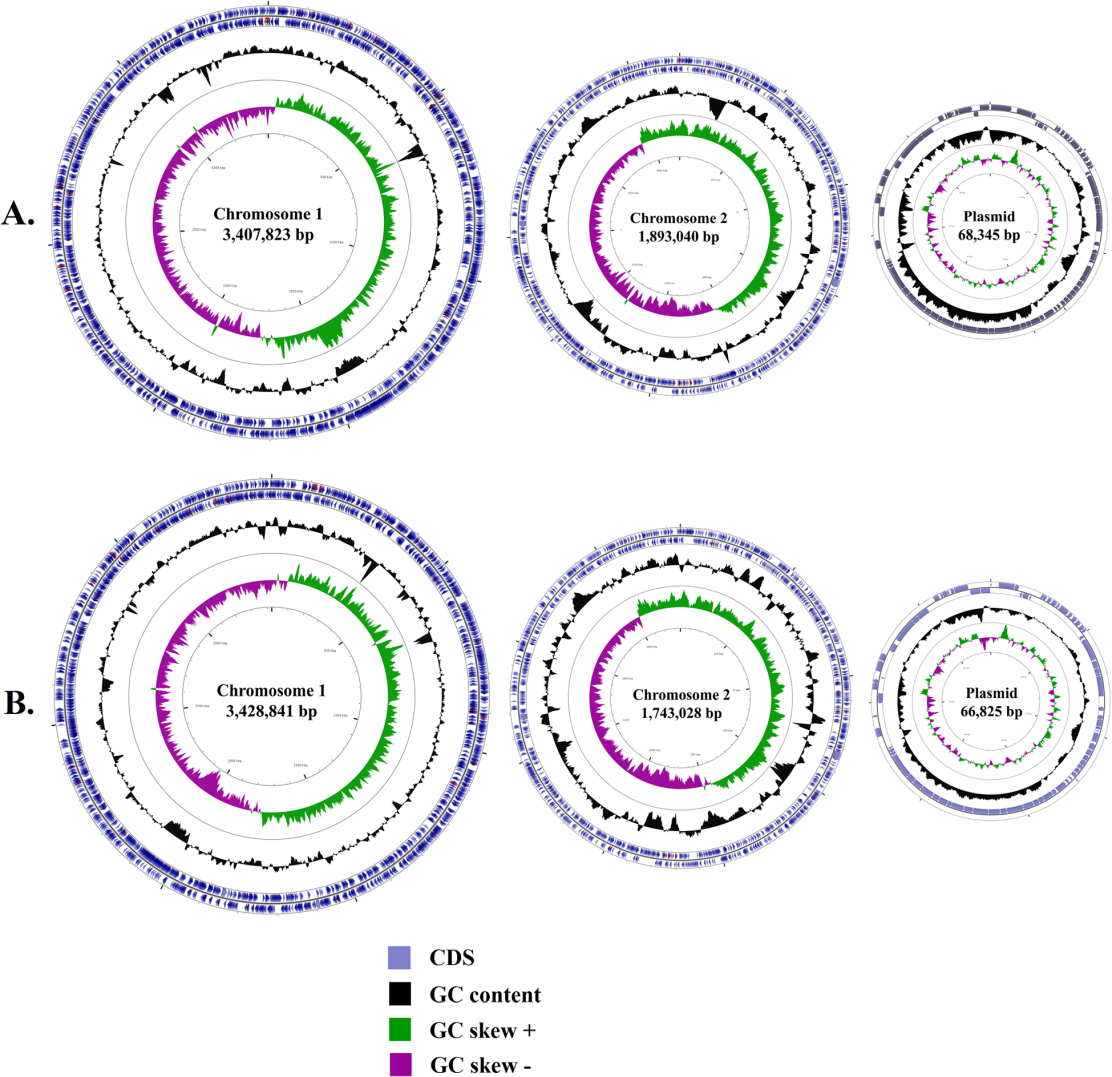
Circular genome representation of the VP_AHPND_ strains A. MSR16 and B. MSR17.

The genomes were assembled into 108 contigs in MSR16 strain and 66 contigs in MSR17 strain. The largest contigs size for MSR16 strain was ~ 1.9 Mbp; and ~ 1.7 Mbp for MSR17 strain. The total GC content was 45.09 and 45.19% for MSR16 and MSR17 strains, respectively. The total genome size of MSR16 was ~ 5.4 Mbp; and ~ 5.2 Mbp for MSR17. MSR16 was found comprised of two circular chromosomes with a length of ~ 3.4 Mbp, ~ 1.8 Mbp while the genome of MSR17 was comprised of similar two circular chromosomes with a length of ~ 3.4 Mbp, ~ 1.7 Mbp. Both MSR16 and MSR17 contain a plasmid with a length of ~ 68 Kbp and ~ 66 Kbp, respectively (Fig. 2). Comparing the genomes, it was observed that chromosome 2 of MSR16 strain has an extra ~100Kb region. More information about MSR16 and MSR17 genomes are given in Table 1.

**Table 1:** Summary of the assembled genomes of two strains (MSR16 & MSR17) of AHPND positive *V. parahaemolyticus*

Features	VP_AHPND_ MSR16	VP_AHPND_ MSR17
Contigs	108	66
Largest contigs	1,892,806	1,742,619
Total length	5,393,740	5,241,592
GC (%)	45.09	45.19
CDS	4909	4689
Gene	5090	4854
tRNA	119	109
misc_RNA	51	45
rRNA	10	10
tmRNA	1	1

The plasmid of MSR16 contains total 87 genes of which 58 genes are hypothetical protein (67%), 5 repeat regions (6%), 7 conjugative transfer proteins (8%), 3 mobile element protein (3%), 2 antirestriction protein (2%), 2 toxin genes (*pir*A and *pir*B) and 10 other genes (11%). The plasmid of MSR17 contains total 88 genes of which 57 genes are hypothetical protein (65%), 6 repeat regions (7%), 7 conjugative transfer proteins (8%), 3 mobile element protein (3%), 2 antirestriction protein (2%), 2 toxin genes (*pir*A and *pir*B) and 11 other genes (13%).

**Fig. 3:**
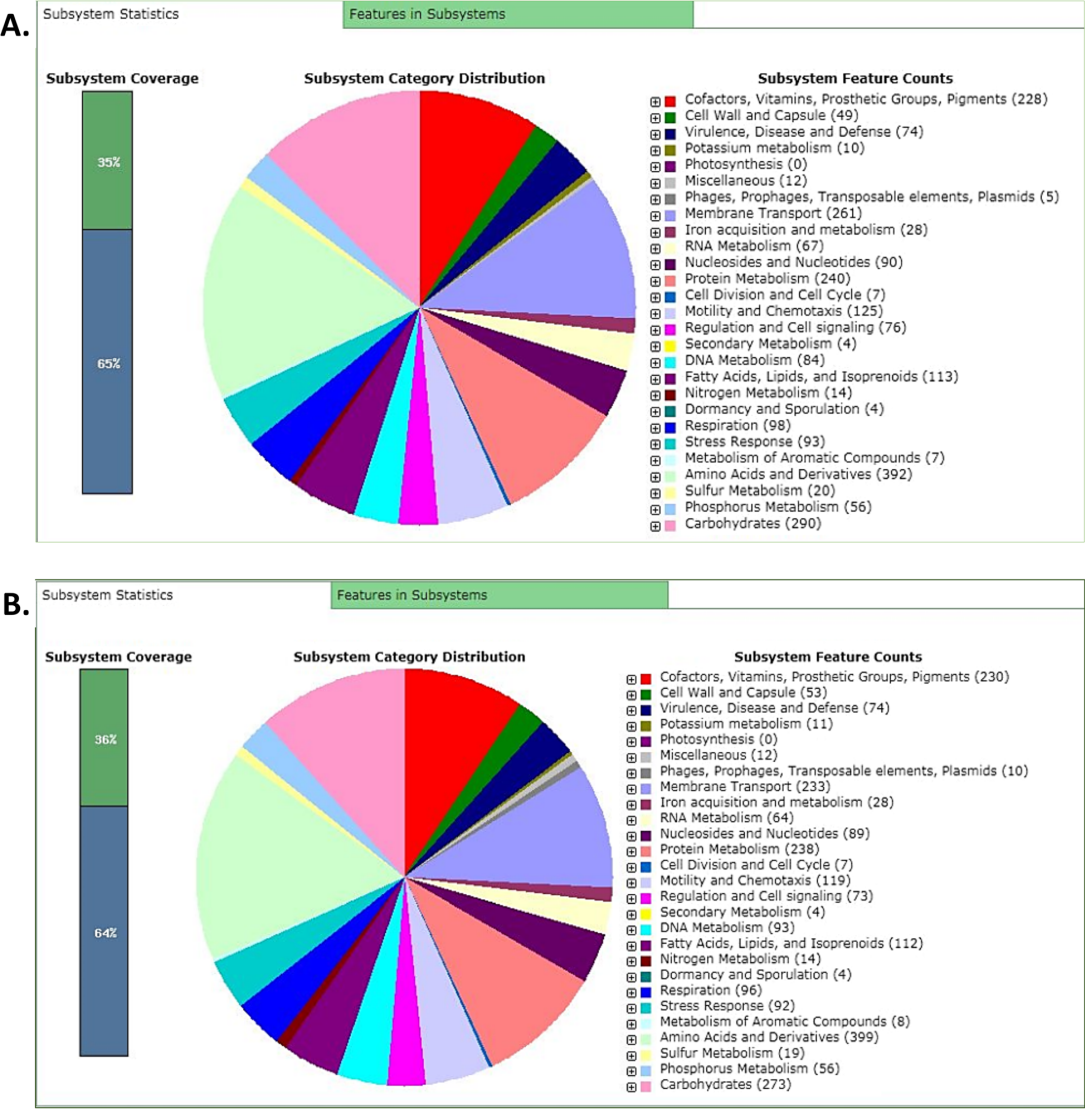
RAST server predicted subsystem categories for AHPND positive *V. parahaemolyticus* strains A. MSR16 and B. MSR17

**Fig. 4:**
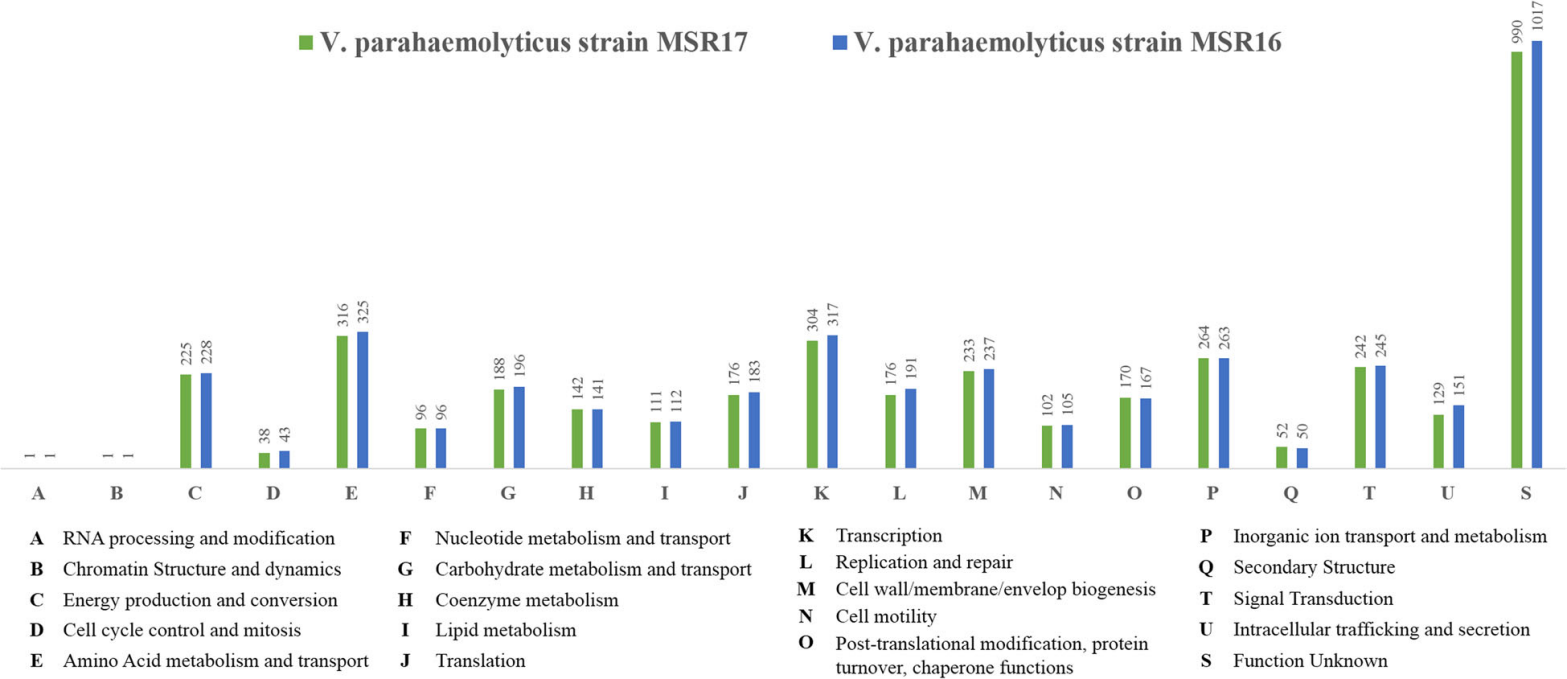
COG classification of the predicted genes in VP_AHPND_ strains MSR16 and MSR17

Out of the RAST server predicted 406 subsystems, MSR16 strain possesses 74 responsible for virulence, disease, and defense; five for phages, prophages, transposable elements and plasmids; 28 for iron acquisition and metabolism; and 125 for motility and chemotaxis. While out of the predicted 403 subsystems, MSR17 strain contained 74 responsible for virulence, disease and defense; 10 for phages, prophages, transposable elements and plasmids; 28 for iron acquisition and metabolism; and 119 for motility and chemotaxis (Fig. 3). These particular subsystems are the hallmarks for the pathogenicity and both strains were found to have almost similar amounts of factors across their genomes. The number of genes associated with the general COG functional categories for both strains is provided in (Fig. 4). Both strains are found to possess an equivalent number of genes associated with those categories.

MSR16 and MSR17 strains have average nucleotide identity values of 98.57% with *V. parahaemolyticus* strain M1–1 and 98.65% with *V. parahaemolyticus* strain 13-306D/4 respectively; they also have an average of 95% ANI values with other AHPND positive strains (Additional file 1). Strains MSR16 and MSR17 have 1403 and 1228 hypothetical genes respectively, whose functional prediction can provide more insights into its pathogenicity and other functional pathways. 144 and 94 unique genes were found in strain MSR16 and MSR17 respectively which are uniquely predicted only for one strain (Additional file 2). MSR17 strain contains unique genes for zona occludens toxin, several transposition proteins, integrase, recombinases, etc.; whereas MSR16 strain has genes for several conjugative transfer related proteins, bacteriocin immunity proteins, etc. Both strains are predicted to have some exclusive genes for diverse metabolic pathways.

### Virulence and antimicrobial resistance genes

Most common 9 virulence factor classes involved in- adherence, antiphagocytosis, enzyme, chemotaxis and motility, iron uptake, quorum sensing, secretion system, toxin, immune evasion were found in the MSR16, while MSR17 possess 8 of these such factors except the factors involved in immune evasion; also few genes in these classes of factors were found absent in these strains (Additional file 3). The major virulence factors of *V. parahaemolyticus* are thermostable direct hemolysin (*tdh*) [17], TDH-related hemolysin (*trh*) [18] and two type III secretion systems (T3SS1 and T3SS2) [19]. *tdh* and *trh* both genes were not found in these strains but the thermolabile hemolysin (*tlh*) gene was found. Between two types of T3SS, only the T3SS1 type was found in MSR16 and MSR17 strain. Both strains possess the plasmid-borne *pir*A and *pir*B toxins.

Antibiotic resistance genes were predicted against β-lactam, fluoroquinolone, tetracycline, macrolide and cephalosporin antibiotics in MSR16; and MSR17 strain has similar resistance genes except for cephalosporin (Additional file 4). Six and two probable prophage regions were found in MSR16 and MSR17 strains, respectively.

Strains MSR16 and MSR17 have approximately 39 and 27 genomic islands (GI) regions respectively (Additional file 5). In strain MSR16, toxin-antitoxin systems like YoeB-YefM, Doc-Phd; antibiotic resistance proteins like FosA (Fosfomycin resistance protein); components of type-I, type-VI secretion systems, etc. are found in those genomic islands. Genomic islands of strain MSR17 contain toxin-antitoxin systems like HipA-HipB, YoeB-YefM; type-I, type-III secretion systems; Multidrug resistance efflux pump; several phage and transposon related proteins, etc. (Additional file 6).

PathogenFinder tool [20] predicted an overall probability of 0.868 for MSR16 and 0.871 for MSR17 for becoming potential human pathogen, so there is a very high risk of spreading these strains into the human food chain and causing human diseases, as several environmental strains of *V. parahaemolyticus* were found to cause cytotoxicity to human gastrointestinal cells even in the absence of *tdh* and/or *trh* genes [21].

### Phylogenetic relationship based on 16S rRNA genes of different AHPND positive *V. parahaemolyticus* strain

**Fig. 5:**
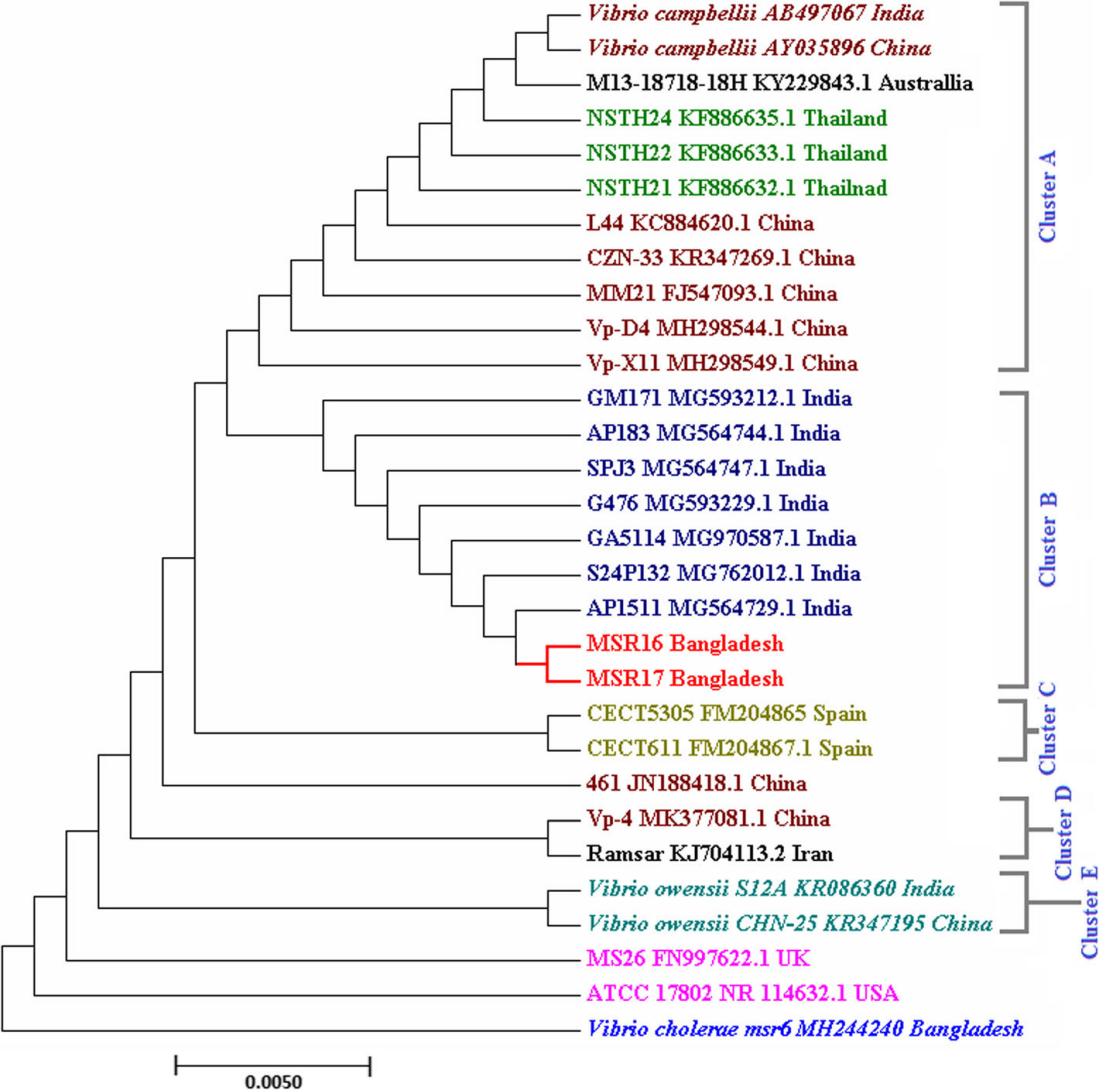
Phylogenetic relationship of 16S rRNA genes of different VP_AHPND_ strains including MSR16 and MSR17 from Bangladesh

A total of 30 strains were selected for establishing a phylogenetic relationship based on the 16S rRNA gene sequence (Fig. 5). The tree includes 25 *V. parahaemolyticus* (including MSR16 and MSR17), two *V. campbellii* and two *V. owensii* strains that were responsible for the AHPND outbreak in recent years in different regions of the world. *V. cholerae* was used for outgroup comparison. In this phylogenetic tree, these strains were distributed in 5 major clusters (Fig. 5).

Most Chinese and Thai strains are found in cluster A. Both of our studied strains (MSR16 and MSR17) located at same cluster B and were closely related with one of the Indian strain AP1511 indicating that the mutation and evolutionary pattern of MSR16 and MSR17 strains might be analogous to this Indian strain. The two Spanish *V. parahaemolyticus* strains separately made cluster C. The strains including Vp-4 MK377081.1 China, Ramsar KJ704113.2 Iran belong to separate cluster D. Besides, two AHPND positive *V. owensii* strains were located at separate cluster E. *V. cholerae* (msr6) strain was distantly related with our studied strains.

### Phylogenetic relationship based on housekeeping genes of different AHPND positive *V. parahaemolyticus*

**Fig. 6:**
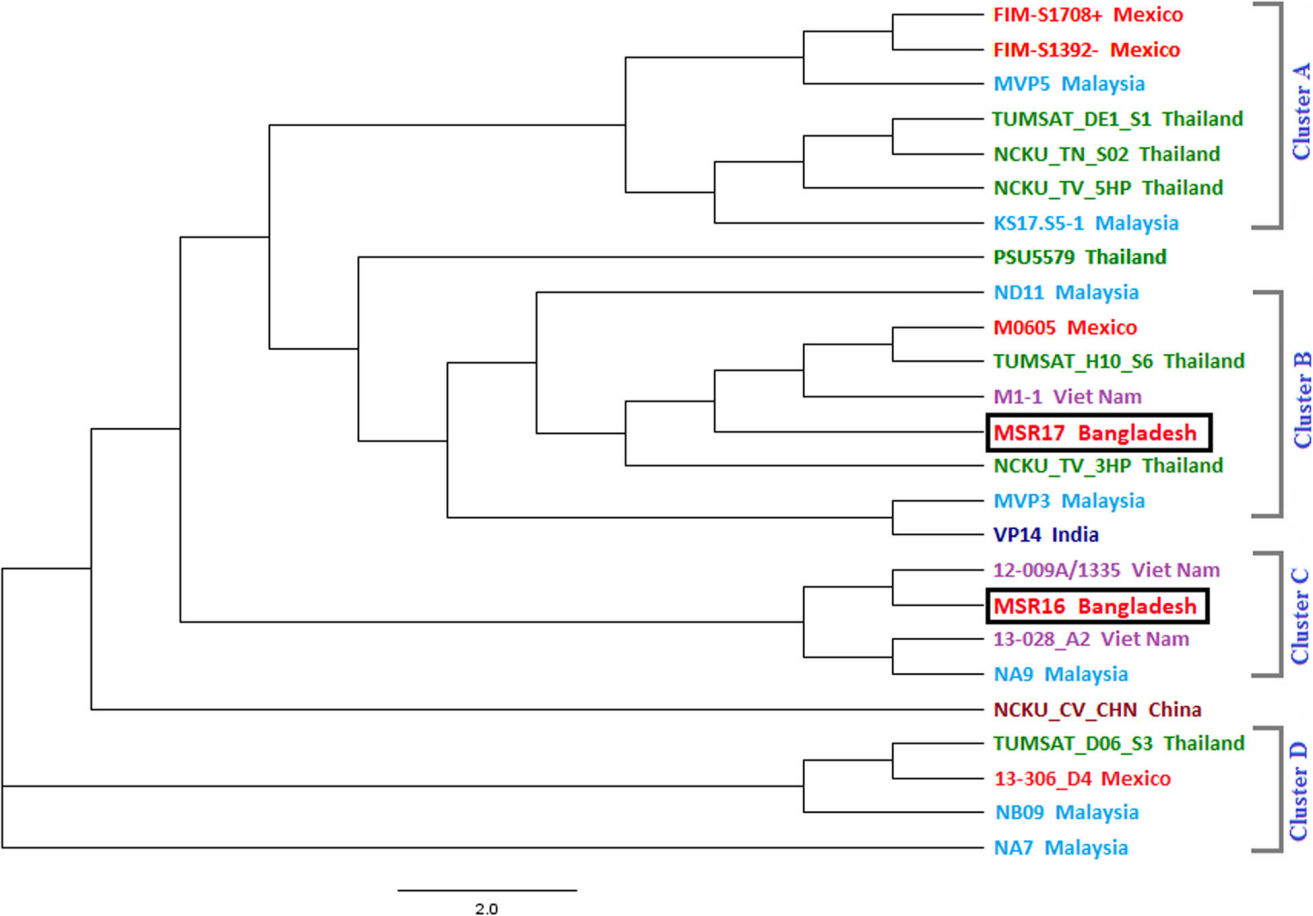
Phylogenetic relationship of using 7 housekeeping genes (*dna*E, *dtd*S, *gyr*B, *pnt*A, *pyr*C, *rec*A, *tna*A) of different VP_AHPND_ strains including MSR16 and MSR17 from Bangladesh

A total of 25 strains were selected for establishing a phylogenetic relationship based on common housekeeping genes (Fig. 6) including (*dna*E, *dtd*S, *gyr*B, *pnt*A, *pyr*C, *rec*A, *tna*A). The 16S rRNA gene was not included because a separate phylogenetic relationship was established based on it. The strains M0605 Mexico, TUMSAT-H10-S6 Thailand, NCKU-TV-3HP Thailand, MSR17 Bangladesh, M1–1 Vietnam, MVP3 Malaysia and VP14 India strain located at same cluster B (Fig. 6). The strains 12-009A/1335 Vietnam, MSR16 Bangladesh, 13–028-A2 Vietnam, and NA9 Malaysia strains located at the same cluster C (Fig. 6).

The phylogenetic tree showed that the MSR16 strain was closely related to the 12-009A/1335 Vietnam strain which maintains an antibacterial type VI secretion system with versatile effector repertoires [22] suggesting that MSR16 strain may have originated from Vietnam. MSR17 strain was closely related to the M1–1 Vietnam strain signifying that MSR17 strain might evolve from M1–1 Vietnamese strain. Kumar et al. (2018) reported that M1–1 strain causes a mild form of shrimp AHPND infection [23]. Compared to other virulent strains, the M1–1 genome was reported to have gained a few additional genes and lost several other genes, which may have resulted in the reduced virulence of this strain [23].

The tree also shows that MSR16 strain arises earlier than MSR17 strain. NA7 Malaysia strain belonged to an independent lineage and distantly related to our studied strains (MSR16 and MSR17) signifying that features from this strain might be dispersed to MSR16 and MSR17 strains.

### ANI (average nucleotide identity) tree of different AHPND positive *V. parahaemolyticus* strain

**Fig. 7:**
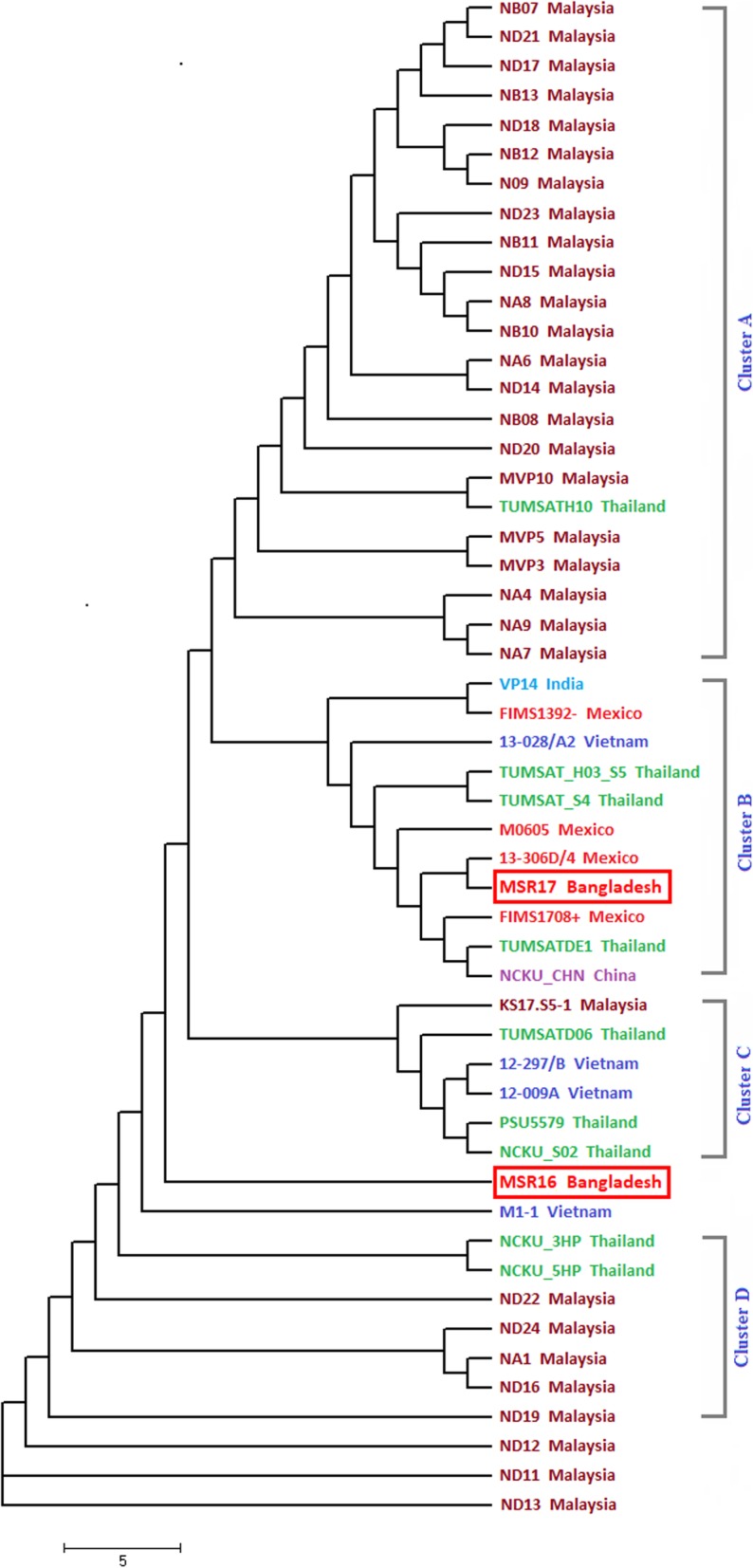
ANI tree of different VP_AHPND_ strains including two VP_AHPND_ strains MSR16 and MSR17 from Bangladesh

A total of 52 genomes of AHPND positive *V. parahaemolyticus* strain including MSR16 and MSR17 were selected for calculating the average nucleotide identity (ANI) (Fig. 7). The ANI tree clearly shows that MSR16 strain belonged to an independent lineage and indicating this strain may have evolved earlier than MSR17. The reason for belonging to an independent lineage might be the presence of an extra ~ 200 kb sequence in the genome. The strain MSR17 was closely related to 13–306-D4 Mexico strain signifying that the average nucleotide identity (ANI) of MSR17 is comparable to this Mexican strain as well as some Thai strains located in cluster B (Fig. 7).

The strains ND11 Malaysia and ND13 Malaysia belonged to an independent lineage as well as distantly related to our studied strain (MSR16, MSR17) indicating that these two strains’ genome sequence might be dispersed to MSR16 and MSR17 strain.

### SNP tree of different AHPND positive *V. parahaemolyticus*

**Fig. 8:**
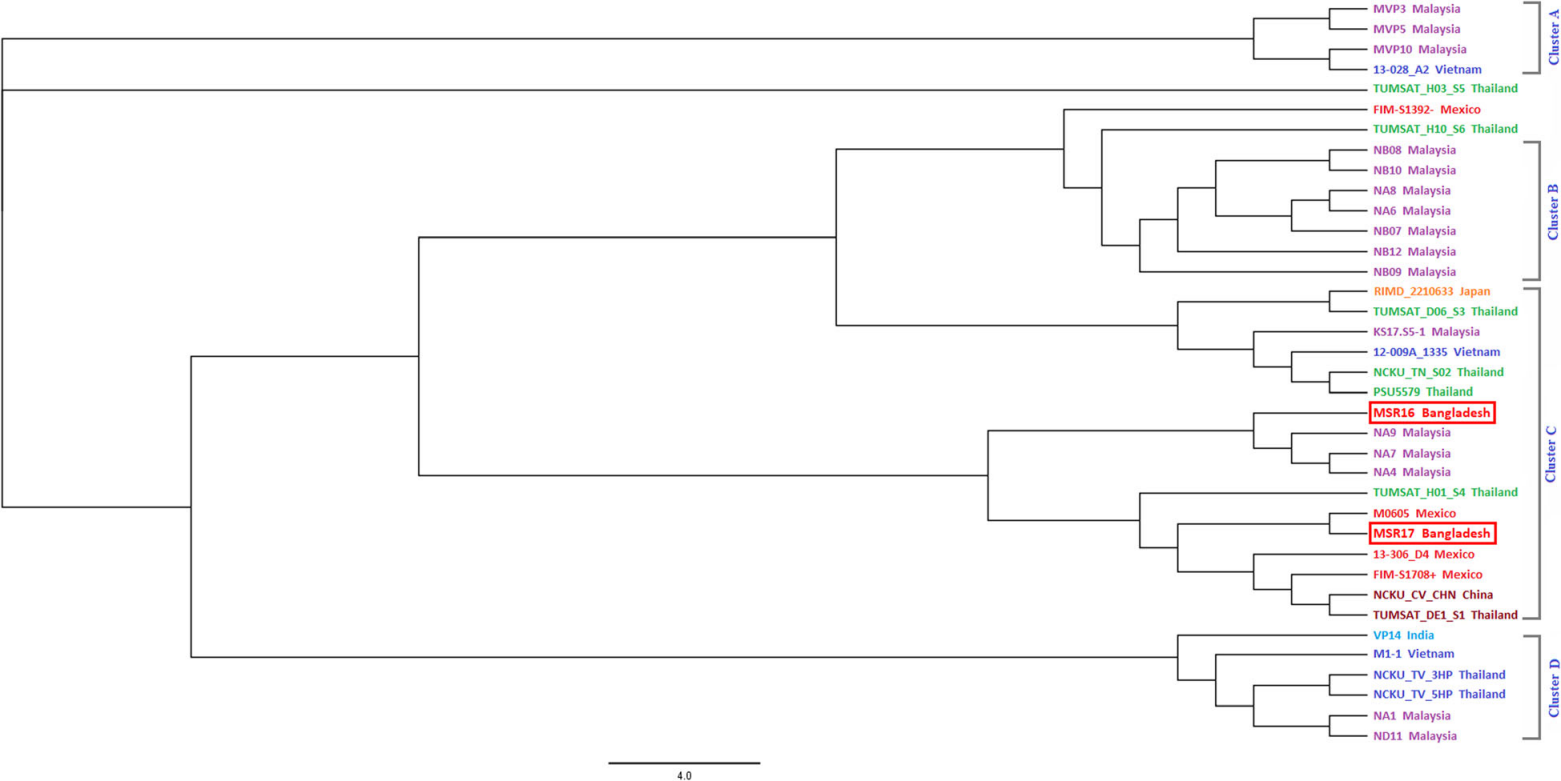
SNP tree of different VP_AHPND_ strains including two VP_AHPND_ strains MSR16 and MSR17 from Bangladesh

A total of 37 genomes of AHPND positive *V. parahaemolyticus* strain including MSR16 and MSR17 were selected for establishing a SNP based relationship (Fig. 8). The strains MSR16 Bangladesh and NA9 Malaysia were closely related and located at the same cluster C (Fig. 8) indicating that the mutation and evolutionary pattern of MSR16 might be comparable to Malaysian strains. NA9 strain was extracted from Malaysian aquaculture pond water which causes AHPND in shrimp and impacting Malaysian shrimp aquaculture. While strains M0605 Mexico and MSR17 Bangladesh were closely related and located at the same cluster C (Fig. 8) indicating that the mutation and evolutionary pattern of MSR17 might be analogous to the Mexican strain. Five iron acquisition systems (hemin, enterobactin, vibrioferrin, and two TonB), 7 secretion systems (two T2SS, one T3SS, two T2/4SS, and two T6SS) and 14 different toxin genes that are involved in the pathogenicity mechanisms were found in both chromosomes of *V. parahaemolyticus* strain M0605 [11]. Gomez-Gil et al. (2014) also detected four plasmids in the M0605 strain’s genome [11]. Strain TUMSAT-H03-S5 Thailand strain belonged to an independent lineage. This strain’s mutation and evolutionary pattern might disperse to MSR16 and MSR17.

### Phylogenetic relationship of identified plasmids found in the AHPND related isolates

**Fig. 9:**
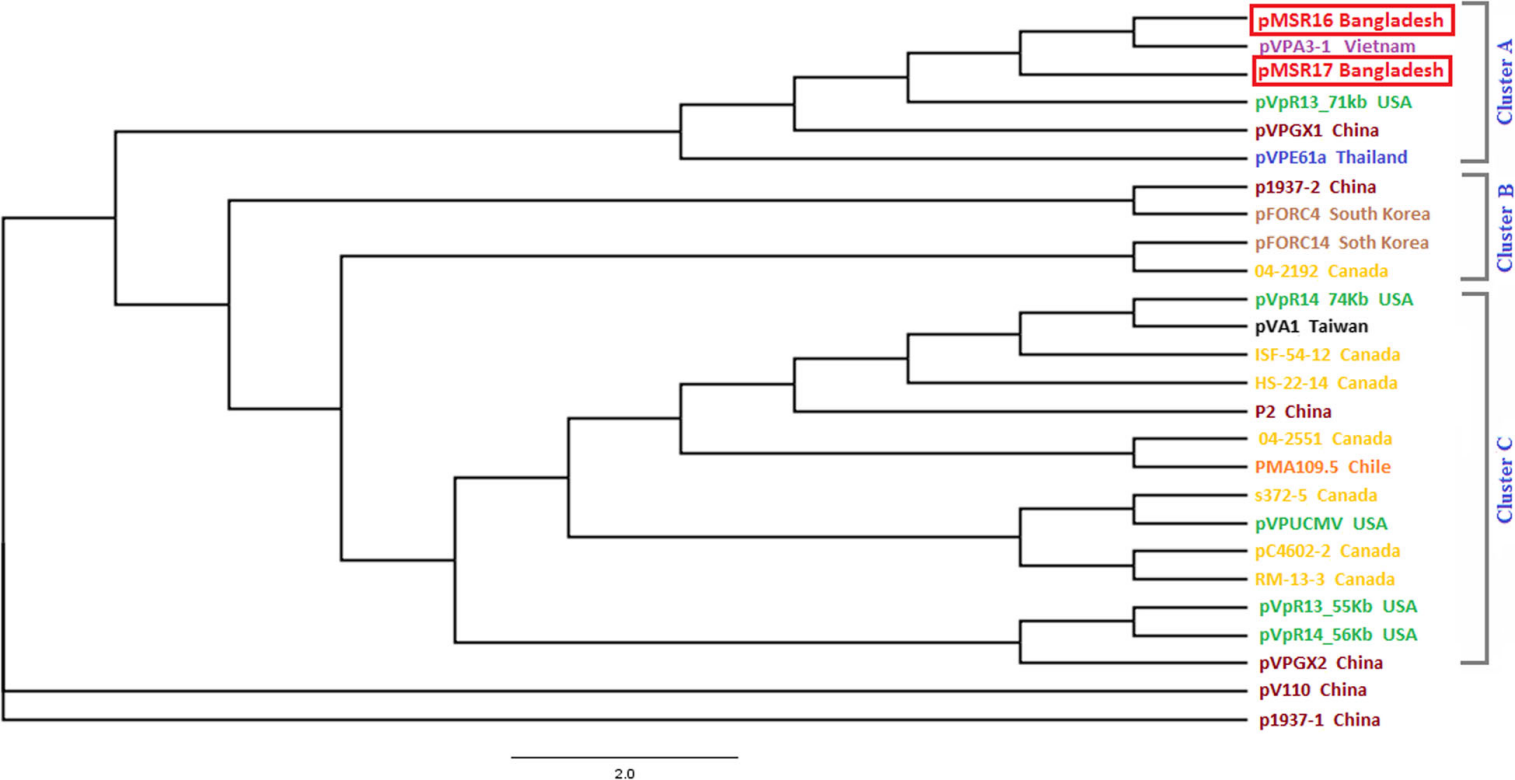
Phylogenetic relationship based on plasmid sequences from VP_AHPND_ isolates including two VP_AHPND_ strains plasmid pMSR16 and pMSR17 from Bangladesh

A total of 26 *V. parahaemolyticus* isolates plasmid including pMSR16 and pMSR17 were selected for establishing the phylogenetic relationship among the AHPND positive *V. parahaemolyticus* plasmid (Fig. 9). Six plasmids including pMSR16 Bangladesh, pVPA3–1 Vietnam, pMSR17 Bangladesh, pVpR13-71Kb USA, pVPGX1 China, pVPE61a Thailand, were located at Cluster A (Fig. 9).

The phylogenetic tree showed that pMSR16 and pMSR17 were closely related to pVPA3–1. The plasmid pVPA3–1 is a Vietnam strain and its accession no. is NC_025152.1. Han et al. (2015) reported that AHPND positive *V. parahaemolyticus* strain 13–028/A3 [9] possess this 69 kb plasmid pVPA3–1 which has 92 open reading frames that encode mobilization proteins, replication enzymes, transposases, virulence-associated proteins, and proteins similar to Photorhabdus insect-related (*Pir*) toxins. The plasmid pV110-KY498540.1 China and p1937–1-NZCP022245.1 China belonged to an independent lineage respectively. These two strains might acquire plasmids from different sources. These two plasmids are also distantly related to our studied plasmids pMSR16 and pMSR17 indicating that they might not have originated from Chinese strains.

## Discussion

*Penaeus monodon* is one of the most important shrimp species that has widely been used for farming in many tropical countries. With the intensification of shrimp farming worldwide, new pathogens are seen to emerge frequently. A variety of microorganisms, such as the White Spot Syndrome Virus (WSSV), *Vibrio* spp. and Taura Syndrome Virus (TSV) has been constantly posing devastating threats to the sustainability of the shrimp farming industry over the years [24]. Since the very first outbreak occurred in China in 2009, because of its rapid deadly effects in the early developmental stages of shrimp, the AHPND/EMS has spread throughout the globe and caused huge economic losses [25].

Recent studies showed that various AHPND positive *V. parahaemolyticus* strains possess the Photorhabdus insect-related (*Pir*) toxin-like genes and these genes (*pir*A and *pir*B like) are likely to be the primary virulence factor in these strains [9]. These gene products are found to be crucial in developing the AHPND in cultured shrimp [26]. The disease has spread and caused major economic losses in Asia as well as in the Americas, and most recently in Texas, USA [27–29]. The *pir*AB region in *V. parahaemolyticus* R13 and R14 strains is encoded on the pVpR13-71Kb and pVpR14-74Kb plasmids, respectively [26, 30]. In *V. parahaemolyticus* R13 strain, the promoter region upstream of *pir*A, the entire open reading frame (ORF) of *pir*A, and part of the 5′ end of the *pir*B ORF were absent and this strain was found avirulent [26]. A particular *V. parahaemolyticus* strain’s capability of causing AHPND depends on the presence of plasmid-borne binary toxins *Pir*AVp and *Pir*BVp [8].

*V. parahaemolyticus* is mostly found in aquatic environments, like- sediments, plankton, and aquatic animals [25]. From the phylogenetic analysis, it has been found out that all AHPND related isolates could be undoubtedly segregated into distinct clusters, where each cluster is specific for a distinctive region [26].

In this study, the genomes of both MSR16 and MSR17 strains contain a plasmid of ~ 69 Kbp. The plasmid of MSR16 contains a total of 87 genes and MSR17 contains a total of 88 genes. Both of the plasmids carry *Pir*A and *Pir*B genes which are responsible for AHPND disease. The length of *pir*A gene was 336 bp (starts at 64,962 bp and stops at 65,297 bp) and the length of *pir*B gene was 1317 bp (starts at 65,310 bp and stops at 66,626 bp) in MSR16. On the other hand, the length of *pir*A gene was 336 bp (starts at 63,108 bp and stops at 63,443 bp) and the length of *pir*B gene was 1317 bp (starts at 63,456 bp and stops at 64,772 bp) in MSR17.

Antibiotic resistance mechanisms can be transmitted from resistant bacteria to other bacteria through the exchange of its naturally occurring resistance genes [31]. It can be observed that both strains possess resistance genes for efflux mechanisms and antibiotic modification which supports our previously reported antibiogram data [6, 32]. While comparing to the experimental results, the presence of several predicted resistance genes against some antibiotics was found in which they are currently either sensitive or have an intermediate response, which means these strains can gain significant antibiotic resistance in the nearest future.

In the present study, the phylogenetic analysis of MSR16 and MSR17 strain was done in several ways such as the construction of trees based on the 16S rRNA gene, common housekeeping genes excluding 16S rRNA, whole plasmid sequences, SNP and average nucleotide identity (ANI).

The phylogenetic tree based on 16S rRNA showed our studied strains MSR16 and MSR17 located at the same cluster and were closely related with one of the Indian strain AP1511 indicating that the mutation and evolutionary pattern of MSR16 and MSR17 strains might be analogous to the Indian strain. The phylogenetic tree based on common housekeeping genes showed that MSR16 strain was closely related to 12-009A/1335 Vietnam and MSR17 strain was closely related to the M1–1 Vietnam strain signifying that evolution of both strains might be from Vietnam.

Analyzing the SNP tree, we have found that MSR16 strain is closely related to three Malaysian strains indicating that the mutation and evolutionary pattern of MSR16 might be comparable to these Malaysian strains. On the other hand, MSR17 strains are closely related to the M0605 Mexico strain indicating that the mutation and evolutionary pattern of MSR17 might be analogous to the Mexican strain.

The ANI tree depicts a diversified pattern for strain MSR16 as it was found occupied in an independent lineage, whereas strain MSR17 was found closely related to Mexican strain 13–306-D4.

The plasmid sequence based phylogenetic tree showed that pMSR16 and pMSR17 were closely related to pVPA3–1. The plasmid pVPA3–1 is a Vietnam strain and its accession no is NC-025152.1. Both plasmids carry the causative agent *pir*A and *pir*B gene of AHPND in their sequence. The plasmid of two studied strains might have evolved from Vietnam. From the above explanations, it can be said that the *V. parahaemolyticus* (AHPND outbreaks) have multiple origins.

## Conclusion

In this study, we report the ~ 5.4 Mbp and ~ 5.2 Mbp genome sequences of *V. parahaemolyticus* strains MSR16 and MSR17 having distinct virulence factors for causing the outbreaks in Bangladesh. Complete resequencing of these genomes of AHPND causing strains MSR16 and MSR17 should provide genomic insights into the pathogenicity and virulence mechanisms of VP_AHPND_. Additional comparative genomics and phylogenetic studies of these two strains may provide understandings of their emergence, spreading patterns so that future outbreaks can be predicted. Also, with the help of different genome sequences collected from outbreaks around the world along with our reported sequences, novel vaccines or drug targets can be identified to tackle any future outbreaks in shellfishes and to reduce the chances of getting these strains introduced in the human food chain to prevent potential health hazards.

## Methods

### Culturing of *V. parahaemolyticus* strains and molecular identification

*V. parahaemolyticus* strains from the previous study (MSR16 and MSR17 strains were isolated from the infected shrimps collected from Morrelganj and Rampal upazila, Bagerhat district of Bangladesh, respectively) [6, 32] were inoculated in Tryptone soy broth (TSB) with 2% salt. The bacteria grow in the TSB were streaked on TCBS agar plate. From TCBS agar plate the bacterial isolates were re-streaked on ChromAgar *Vibrio* medium (CHROMagar, Paris-France). The bacterial isolates were further streaked on Tryptone soya agar (TSA) with 2% salt to obtain pure isolates. To support the vigorous growth of *V. parahaemolyticus* strain, Luria Bertani (LB) broth was used with 2% salt. Total genomic DNA was extracted using DNeasy Blood & Tissue Kit (Qiagen, Hilden, Germany) following the manufacturer’s protocol. The DNA quality was quantified using NanoDrop spectrophotometer (Thermo Scientific, Waltham, MA, USA). Polymerase chain reaction (PCR) was used for the partial amplification of 16S rRNA, *ldh*, AP3 and AP4 genes for the molecular identification of suspected AHPND positive *V. parahaemolyticus* strains.

### Sequencing and assembly

A genomic library was constructed and employed for 150 bp paired-end whole-genome sequencing using an Illumina MiSeq platform (Illumina, San Diego, CA, USA). An in house pipeline was built to perform the whole assembly process which performed i) Adapter and low-quality base trimming using Trimmomatic v0.38 [33] using several parameters; ii) Generation of QC reports of trimmed and untrimmed data using FastQC v0.11.7 [34]; iii) Genome Assembly using the trimmed and untrimmed data by SPAdes v3.10 [35] in both general and plasmid mode utilizing different k-mer combinations; iv) Assembly polishing using Pilon v1.22 [36]; v) Determination of the quality and coverage of the assemblies using Quast v5.0.2 [37]; vi) Scaffolding into chromosomes and plasmids by MeDuSa v1.6 [38]. Genomic scaffolds of these two strains were compared using Mauve 2.4.0 [39].

### Gene prediction and annotation

Genome annotation was performed by Prokka v1.12 [40], Glimmer v3.02 [41], RASTtk v1.3.0 [42] and tRNA, rRNA annotation was done using Barrnap v0.6, tRNAscan-SE v2.0 [43]. Average nucleotide identity of 50 different AHPND causing *V. parahaemolyticus* strains were calculated using Pyani v0.2.7 [44].

### Analysis of virulence, antibiotic resistance genes, and others

Virulence factors were searched using VFanalyzer [45]. ResFinder [46], ARG-ANNOT (Antibiotic Resistance Gene-ANNOTation) [47] and CARD tools [48] were used to search antibiotic resistance genes. Prophage sequences were searched respectively by PHASTER [49]. COG (Clusters of orthologous groups) classification of the genes was achieved by eggNOG-mapper v1 [50]. Genomic islands were predicted using the Islandviewer tool [51].

### Phylogenetic analysis and genome comparison

A phylogenetic tree was constructed based on 16S rDNA sequences of several AHPND causing *V. parahaemolyticus* strains from around different parts of the world using MEGA 7.0 software [52]. The evolutionary history was inferred by using the Maximum Likelihood method with 100 bootstraps based on the best model fit for this dataset analyzed by MEGA, in this case, the Tamura-Nei (TN) [53] model which had the lowest BIC score.

Housekeeping genes of 25 different *V. parahaemolyticus* strains were obtained from MLST 2.0 server (https://cge.cbs.dtu.dk/services/MLST/) using the assembled genomes of those strains. Gene sequences were extracted and concatenated using in house shell scripts and a Neighbor-joining tree with 100 bootstraps based on Kimura two-parameter (K2P) substitution model was constructed using MEGA 7.0 software [52].

SNP based NJ-tree was constructed with genomes of 37 different AHPND positive *V. parahaemolyticus* strains using the Parsnp v1.2 tool [54]. Average nucleotide identity (ANI) based tree was constructed from our generated ANI value matrix (Additional file 1) using PHYLIP package [55]. Twenty-six plasmids from several AHPND positive *V. parahaemolyticus* strains were aligned and a NJ-tree was constructed with 50 bootstraps using MAFFT v7 [56] utilizing the Jukes-Cantor (JK) substitution model.

### Quality assurance

16S rRNA genes of *V. parahaemolyticus* strain MSR16 and MSR17 were predicted from the annotation pipeline and also from the BLAST [57] search of the PCR amplified partial sequences of both strains’ 16S rRNA genes.

## Supplementary information

**Additional file 1. **Matrix representation of ANI values of strains MSR16 and MSR17 compared to other AHPND positive *V. parahaemolyticus* strains.

**Additional file 2.** Unique genes predicted in VP_AHPND_ strains MSR16 and MSR17.

**Additional file 3.** Virulence property of VP_AHPND_ strains MSR16 and MSR17 predicted by VFAnalyzer.

**Additional file 4.** Predicted antibiotic resistance genes in MSR16 and MSR17 strains of VP_AHPND_.

**Additional file 5. **Number of Genomic islands (GIs) predicted for AHPND positive *V. parahaemolyticus* strains MSR16 and MSR17.

**Additional file 6.** List of genes found in the Genomic islands (GIs) of VP_AHPND_ strains MSR16 and MSR17.

## Data Availability

The draft sequences of both *Vibrio parahaemolyticus* strains MSR16 and MSR17 can be found in NCBI Bioproject ID PRJNA505599.

## Abbreviations

AHPND: Acute hepatopancreatic necrosis disease
ANI: Average Nucleotide Identity
COG: Clusters of orthologous groups
EMS: Early mortality syndrome
GI: Genomic Island
*pir*: Photorhabdus insect-related
SNP: Single nucleotide polymorphism
T3SS: Type III secretion system
WGS: Whole genome sequencing
