# Supplementary material for: Genomic and evolutionary features of two AHPND positive Vibrio parahaemolyticus strains isolated from shrimp (Penaeus monodon) of south-west Bangladesh
Source: BMC Microbiol. 2019 Dec 3;19:270. doi: 10.1186/s12866-019-1655-8 (PMC6889531; doi:10.1186/s12866-019-1655-8)
Supplement: Supplementary file 2 — Additional file 2. Unique genes predicted in VPAHPND strains MSR16 and MSR17. [file 12866_2019_1655_MOESM2_ESM.docx]

**Additional file 2: Unique genes predicted in VPAHPND strains MSR16 and MSR17.**

| Serial Number | Genes exclusive in MSR 16 | Genes exclusive in MSR 17 |
| --- | --- | --- |
| 1 | Transcriptional regulator | FIG01200653: hypothetical protein |
| 2 | Uncharacterized protein Caur_1846 | FIG01201645: hypothetical protein |
| 3 | Error-prone repair protein UmuD | Prophage Lp2 protein 6 |
| 4 | Error-prone, lesion bypass DNA polymerase V (UmuC) | FIG01200466: hypothetical protein |
| 5 | FIG01199668: hypothetical protein | 2-oxoglutarate/2-oxoacid ferredoxin oxidoreductase, gamma subunit (EC 1.2.7.-) / 2-oxoglutarate/2- oxoacid ferredoxin oxidoreductase, alpha subunit (EC 1.2.7.-) |
| 6 | 2-keto-4-pentenoate hydratase (EC 4.2.1.80) | 2-oxoglutarate/2-oxoacid ferredoxin oxidoreductase, beta subunit (EC 1.2.7.-) |
| 7 | FIG023406: hypothetical protein | putative regulator protein |
| 8 | FIG01199963: hypothetical protein | Zona occludens toxin |
| 9 | FIG01204144: hypothetical protein | Accessory cholera enterotoxin |
| 10 | FIG01056146: hypothetical protein | RstB phage-related integrase |
| 11 | Nudix hydrolase family protein | FIG01202409: hypothetical protein |
| 12 | S-adenosylhomocysteine hydrolase | FIG01205912: hypothetical protein |
| 13 | FIG01199625: hypothetical protein | FIG01204166: hypothetical protein |
| 14 | Structural protein P5 | FIG01201016: hypothetical protein |
| 15 | FIG01203955: hypothetical protein | FIG01203636: hypothetical protein |
| 16 | FIG01204114: hypothetical protein | FIG01204047: hypothetical protein |
| 17 | FIG01203368: hypothetical protein | FIG01202078: hypothetical protein |
| 18 | FIG01199929: hypothetical protein | Putative superfamily I DNA helicases |
| 19 | FIG01202154: hypothetical protein | FIG01203961: hypothetical protein |
| 20 | FIG01201474: hypothetical protein | FIG01201403: hypothetical protein |
| 21 | FIG01204209: hypothetical protein | FIG01201642: hypothetical protein |
| 22 | FIG01199719: hypothetical protein | Type I restriction-modification system, restriction subunit R (EC 3.1.21.3) |
| 23 | FIG01199587: hypothetical protein | Anticodon nuclease |
| 24 | Replication gene A protein | Type I restriction-modification system, specificity subunit S |
| 25 | FIG01203403: hypothetical protein | Uncharacterized NAD(P)H oxidoreductase, YdeQ/YrkL/YwrO family |
| 26 | FIG01203441: hypothetical protein | RNA helicase |
| 27 | FIG01202428: hypothetical protein | Acetyltransferase, GNAT family (EC 2.3.1.-) |
| 28 | IncF plasmid conjugative transfer pilin protein TraA | putative acetyltransferase |
| 29 | IncF plasmid conjugative transfer pilus assembly protein TraL | FIG01201438: hypothetical protein |
| 30 | IncF plasmid conjugative transfer pilus assembly protein TraE | anticodon nuclease |
| 31 | IncF plasmid conjugative transfer pilus assembly protein TraK | Phage integrase family protein |
| 32 | IncF plasmid conjugative transfer pilus assembly protein TraB | Putative transcriptional regulator |
| 33 | IncF plasmid conjugative transfer pilus assembly protein TraV | patatin-related protein |
| 34 | IncF plasmid conjugative transfer pilus assembly protein TraC | Attachment to host cells and virulence |
| 35 | IncF plasmid conjugative transfer protein TrbI | site-specific DNA-methyltransferase, putative |
| 36 | IncF plasmid conjugative transfer pilus assembly protein TraW | enzyme; Degradation of DNA |
| 37 | IncF plasmid conjugative transfer pilus assembly protein TraU | Malonyl CoA-acyl carrier protein transacylase |
| 38 | Unknown | Phage replication protein |
| 39 | IncF plasmid conjugative transfer protein TraN | FIG01206675: hypothetical protein |
| 40 | IncF plasmid conjugative transfer pilus assembly protein TraF | Dolichol-phosphate mannosyltransferase |
| 41 | IncF plasmid conjugative transfer protein TrbB | FIG01204007: hypothetical protein |
| 42 | IncF plasmid conjugative transfer pilus assembly protein TraH | Antitoxin DinJ (binds YafQ toxin) |
| 43 | FIG01067416: hypothetical protein | Glucose 1-dehydrogenase (EC 1.1.1.47) |
| 44 | IncF plasmid conjugative transfer protein TraD | LSU rRNA ## 23S rRNA, large subunit ribosomal RNA |
| 45 | Extracellular deoxyribonuclease Dns (EC 3.1.21.-) | probable membrane protein YPO2863 |
| 46 | IncF plasmid conjugative transfer DNA-nicking and unwinding protein TraI | FIG01201318: hypothetical protein |
| 47 | IncI1 plasmid conjugative transfer putative membrane protein PilT | FIG01201377: hypothetical protein |
| 48 | RelB/StbD replicon stabilization protein (antitoxin to RelE/StbE) | Arginine decarboxylase, catabolic (EC 4.1.1.19) |
| 49 | mRNA interferase RelE | Arginine decarboxylase (EC 4.1.1.19); Ornithine decarboxylase (EC 4.1.1.17); Lysine decarboxylase (EC 4.1.1.18) |
| 50 | transposase for IS1001 element | FIG01204437: hypothetical protein |
| 51 | N-acyl-L-amino acid amidohydrolase (EC 3.5.1.14) | Outer membrane protein H1 |
| 52 | Metallopeptidase, M24 family | Miniconductance mechanosensitive channel MscM |
| 53 | Asp/Glu racemase | ABC transporter, ATP-binding protein (cluster 9, phospholipid) |
| 54 | Diaminopropionate ammonia-lyase (EC 4.3.1.15) | ABC transporter, permease protein (cluster 9, phospholipid) |
| 55 | Outer membrane lipoprotein precursor, OmpA family | Resolvase |
| 56 | FIG01205343: hypothetical protein | COG0582: Integrase |
| 57 | Glycerol dehydrogenase (EC 1.1.1.6) | HigA protein (antitoxin to HigB) |
| 58 | Cytolysin and hemolysin, HlyA, Pore-forming toxin | Bipolar DNA helicase HerA |
| 59 | transcriptional regulator, AraC family | Tn7-like transposition protein A |
| 60 | Replication protein | Transposon Tn7 transposition protein tnsB |
| 61 | Prophage Lp2 protein 4 | Tn7-like transposition protein C |
| 62 | DNA-methyltransferase | Tn7-like transposition protein D |
| 63 | FIG01199650: hypothetical protein | Uncharacterized protein Ctu_08830 |
| 64 | transaldolase( EC:2.2.1.2 ) | ATP-binding protein |
| 65 | putative (L31491) ORF2; putative [Plasmid pTOM9] | Putative predicted metal-dependent hydrolase |
| 66 | putative ORF1 [Plasmid pTOM9] | FIG01202149: hypothetical protein |
| 67 | Transcriptional repressor RcnR | Dca |
| 68 | FIG01205018: hypothetical protein | UDP-N-acetylglucosamine 4,6-dehydratase (EC 4.2.1.135) |
| 69 | Nucleoside-diphosphate-sugar epimerases | Aminotransferase, DegT/DnrJ/EryC1/StrS family |
| 70 | FIG01201374: hypothetical protein | N-acetylneuraminate synthase (EC 2.5.1.56) |
| 71 | FIG01203905: hypothetical protein | 4-amino-6-deoxy-N-Acetyl-D-hexosaminyl-(Lipid carrier) acetyltrasferase |
| 72 | FIG01201881: hypothetical protein | D-glycero-D-manno-heptose 1-phosphate guanosyltransferase |
| 73 | RstR phage-related transcriptional repressor | N-acylneuraminate cytidylyltransferase (EC 2.7.7.43) |
| 74 | Ankyrin | aminotransferase, Class III pyridoxal-phosphate dependent |
| 75 | resolvase, putative | Aliphatic amidase AmiE (EC 3.5.1.4) |
| 76 | FIG01204372: hypothetical protein | Glutamine--fructose-6-phosphate transaminase (isomerizing), glutaminase subunit (EC 2.6.1.16) |
| 77 | Protein ygiW precursor | FIG01202033: hypothetical protein |
| 78 | FIG01205959: hypothetical protein | dTDP-4-dehydrorhamnose reductase (EC 1.1.1.133) |
| 79 | bacteriophage f237 ORF9 | dTDP-4-dehydrorhamnose 3,5-epimerase (EC 5.1.3.13) |
| 80 | FIG01200378: hypothetical protein | Alpha-1, 4-N-acetylgalactosamine transferase PglH (EC 2.4.1.-) |
| 81 | Msl2237 protein | Glycosyltransferase PglI (EC 2.4.1.-) |
| 82 | MazG protein domain | FIG01199943: hypothetical protein |
| 83 | Fosfomycin resistance protein FosA | FIG01045697: hypothetical protein |
| 84 | ADP-ribose 1-phosphate phophatase related protein | FIG01205366: hypothetical protein |
| 85 | MutT/nudix family protein | FIG01202923: hypothetical protein |
| 86 | Cold shock protein of CSP family / Excalibur calcium-binding domain | DnaJ-class molecular chaperone with C-terminal Zn finger domain |
| 87 | ORF2 | FIG01205453: hypothetical protein |
| 88 | OsmC/Ohr family protein | FIG01203507: hypothetical protein |
| 89 | Putative type-I secretion protein | FIG01203383: hypothetical protein |
| 90 | Phosphatidylinositol-specific phospholipase C (EC 4.6.1.13) | FIG01204183: hypothetical protein |
| 91 | Lactaldehyde dehydrogenase involved in fucose or rhamnose utilization (EC 1.2.1.22) | putative short-chain dehydrogenase |
| 91 | L-fuculose phosphate aldolase (EC 4.1.2.17) | thioredoxin-dependent thiol peroxidase |
| 93 | L-fucose mutarotase (EC 5.1.3.29) | FIG01203151: hypothetical protein |
| 94 | L-fuculokinase (EC 2.7.1.51) | Site-specific recombinase, phage integrase family domain protein |
| 95 | L-fucose isomerase (EC 5.3.1.25) @ D-Arabinose ketol-isomerase (EC 5.3.1.3) |  |
| 96 | Fucose permease |  |
| 97 | L-fucose operon activator |  |
| 98 | serine/threonine kinase |  |
| 99 | FIG00847736: hypothetical protein |  |
| 100 | FIG01202705: hypothetical protein |  |
| 101 | FIG01203734: hypothetical protein |  |
| 102 | Putative acetyltransferase |  |
| 103 | Probable acetyltransferase |  |
| 104 | Ketosteroid isomerase-related protein |  |
| 105 | Tricorn protease N-terminal domain-containing protein |  |
| 106 | FIG01201975: hypothetical protein |  |
| 107 | putative PnuC protein |  |
| 108 | GDP-L-fucose synthetase (EC 1.1.1.271); Colanic acid biosynthesis protein wcaG |  |
| 109 | LysR family transcriptional regulator YfiE |  |
| 110 | Cysteine/O-acetylserine efflux protein |  |
| 111 | Cell surface protein |  |
| 112 | Nucleoside permease |  |
| 113 | Prevent host death protein, Phd antitoxin |  |
| 114 | DNA integration/recombination/inversion protein |  |
| 115 | FIG01205024: hypothetical protein |  |
| 116 | conserved hypothetical protein |  |
| 117 | LSU rRNA # 23S rRNA, large subunit ribosomal RNA - 5 prime truncation |  |
| 118 | FIG01200292: hypothetical protein |  |
| 119 | FIG01203580: hypothetical protein |  |
| 120 | bacteriocin immunity protein |  |
| 121 | dTDP-6-deoxy-3,4-keto-hexulose isomerase (EC 5.3.2.3) |  |
| 122 | transferase hexapeptide repeat |  |
| 123 | MaoC-like dehydratase |  |
| 124 | dTDP-3-amino-3,6-dideoxy-alpha-D-galactopyranose transaminase (EC 2.6.1.90) |  |
| 125 | YjbG polysaccharide synthesis-related protein |  |
| 126 | FIG01203002: hypothetical protein |  |
| 127 | Glycosyltransferase (EC 2.4.1.-) |  |
| 128 | FIG01201713: hypothetical protein |  |
| 129 | SSU rRNA # 16S rRNA, small subunit ribosomal RNA - 5 prime truncation - 3 prime truncation |  |
| 130 | UPF0339 protein YegP |  |
| 131 | Epoxyqueuosine (oQ) reductase QueG |  |
| 132 | Phage NinG rap recombination |  |
| 133 | Primosomal protein I |  |
| 134 | LicD family protein |  |
| 135 | Sugar-phosphate cytidylyltransferase |  |
| 136 | polysaccharide pyruvyl transferase |  |
| 137 | Lipopolysaccharide N-acetylmannosaminouronosyl transferase (EC 2.4.1.180) |  |
| 138 | Putative acid amine ligase YjfC |  |
| 139 | Formate dehydrogenase O gamma subunit (EC 1.2.1.2) |  |
| 140 | FIG01200289: hypothetical protein |  |
| 141 | putative resolvase |  |
| 142 | FIG01205557: hypothetical protein |  |
| 143 | FIG01200973: hypothetical protein |  |
| 144 | Dienelactone hydrolase and related enzymes |  |
