# Supplementary material for: Genomic and evolutionary features of two AHPND positive Vibrio parahaemolyticus strains isolated from shrimp (Penaeus monodon) of south-west Bangladesh
Source: BMC Microbiol. 2019 Dec 3;19:270. doi: 10.1186/s12866-019-1655-8 (PMC6889531; doi:10.1186/s12866-019-1655-8)
Supplement: Supplementary file 3 — Additional file 3. Virulence property of VPAHPND strains MSR16 and MSR17 predicted by VFAnalyzer. [file 12866_2019_1655_MOESM3_ESM.docx]

**Additional file 3:** **Virulence property of VPAHPND strains MSR16 and MSR17 predicted by VFAnalyzer.**

| VF class | Virulence factors | Related genes | *V. parahaemolyticus* MSR16 (Prediction) | *V. parahaemolyticus* MSR17 (Prediction) |
| --- | --- | --- | --- | --- |
| Adherence | Accessory colonization factor | acfA | - | - |
|  |  | acfB | - | - |
|  |  | acfC | - | - |
|  |  | acfD | - | - |
|  | Mannose-sensitive hemagglutinin (MSHA type IV pilus) | mshA | orf00616; orf04164 | orf04064; orf04065 |
|  |  | mshB | orf04163 | - |
|  |  | mshC | orf04165 | - |
|  |  | mshD | orf04166 | orf04066 |
|  |  | mshE | orf04159 | orf04060 |
|  |  | mshF | orf04161 | orf04062 |
|  |  | mshG | orf04160 | orf04061 |
|  |  | mshH | orf04152 | orf04052 |
|  |  | mshI | orf04153 | orf04053 |
|  |  | mshJ | orf04154 | orf04054 |
|  |  | mshK | orf04155 | orf04055 |
|  |  | mshL | orf04156 | - |
|  |  | mshM | orf04157 | orf04058 |
|  |  | mshN | orf04158 | orf04059 |
|  | Toxin-coregulated pilus (type IVB pilus) | tcpA | - | - |
|  |  | tcpB | - | - |
|  |  | tcpC | - | - |
|  |  | tcpD | - | - |
|  |  | tcpE | - | - |
|  |  | tcpF | - | - |
|  |  | tcpH | - | - |
|  |  | tcpI | - | - |
|  |  | tcpJ | - | - |
|  |  | tcpN/toxT | - | - |
|  |  | tcpP | - | - |
|  |  | tcpQ | - | - |
|  |  | tcpR | - | - |
|  |  | tcpS | - | - |
|  |  | tcpT | - | - |
|  | Type IV pilus | pilA | orf01876 | - |
|  |  | pilB | orf01875 | orf01694 |
|  |  | pilC | orf01874 | orf01693 |
|  |  | pilD | orf01873 | orf01692 |
|  | The tad locus (Haemophilus) | tadA | orf01084 | - |
|  | Type IV pili (Yersinia) | pilW | orf04931 | orf04678 |
| Antiphagocytosis | Capsular polysaccharide | cpsA | orf01260 | orf00466 |
|  |  | cpsB | orf01261 | orf00465 |
|  |  | cpsC | orf01262 | orf00464 |
|  |  | cpsD | orf01263 | orf00463 |
|  |  | cpsE | orf01264 | orf00462 |
|  |  | cpsF | orf01265 | orf00461 |
|  |  | cpsG | orf01266 | orf00460 |
|  |  | cpsH | orf01267 | orf00459 |
|  |  | cpsI | orf01268 | orf00458 |
|  |  | cpsJ | orf01269 | orf00457 |
|  |  | hp1 | - | - |
|  |  | rmlA | - | - |
|  |  | rmlB | - | - |
|  |  | rmlC | - | - |
|  |  | rmlD | - | orf03284 |
|  |  | wbfB | - | - |
|  |  | wbfC | - | - |
|  |  | wbfT | - | - |
|  |  | wbfU | - | - |
|  |  | wbfV/wcvB | orf02709 | orf03295 |
|  |  | wbfY | - | - |
|  |  | wbjD/wecB | orf02700 | - |
|  |  | wbuB | - | - |
|  |  | wcaJ | - | - |
|  |  | wecA | orf02708 | orf03294 |
|  |  | wecC | - | - |
|  |  | wza | orf01454 | orf00273 |
|  |  | wzb | - | - |
|  |  | wzc | orf01456 | orf00271 |
| Chemotaxis and motility | Flagella | cheA | orf02479 | orf02008 |
|  |  | cheB | orf02478 | orf02009 |
|  |  | cheR | orf03619 | orf03804 |
|  |  | cheV | orf03618 | orf03803 |
|  |  | cheW | orf02475 | orf02012 |
|  |  | cheY | orf02481 | orf02006 |
|  |  | cheZ | orf02480 | orf02007 |
|  |  | filM | orf02492 | orf01995 |
|  |  | flaA | orf03633 | orf03816 |
|  |  | flaB | orf02508 | orf01979 |
|  |  | flaC | - | - |
|  |  | flaD | orf02509 | orf01978; orf03817 |
|  |  | flaE | orf02131 | orf01977 |
|  |  | flaG | orf02507 | orf01980 |
|  |  | flaI | orf02505 | orf01982 |
|  |  | flgA | orf03617 | orf03802 |
|  |  | flgB | orf03620 | orf03805 |
|  |  | flgC | orf03621 | orf03806 |
|  |  | flgD | orf03622 | orf03807 |
|  |  | flgE | orf03623 | orf03808 |
|  |  | flgF | orf03625 | orf03809 |
|  |  | flgG | orf03626 | orf03810 |
|  |  | flgH | orf03627 | orf03811 |
|  |  | flgI | orf03628 | orf03812 |
|  |  | flgJ | orf03629 | orf03813 |
|  |  | flgK | orf03630 | orf03814 |
|  |  | flgL | orf03631 | orf03815 |
|  |  | flgM | orf03616 | orf03801 |
|  |  | flgN | orf03615 | orf03800 |
|  |  | flhA | orf02485 | orf02002 |
|  |  | flhB | orf02486 | orf02001 |
|  |  | flhF | orf02484 | orf02003 |
|  |  | flhG | orf02483 | orf02004 |
|  |  | fliA | orf02482 | orf02005 |
|  |  | fliD | orf02506 | orf01981 |
|  |  | fliE | orf02500 | orf01987 |
|  |  | fliF | orf02499 | orf01988 |
|  |  | fliG | orf02498 | orf01989 |
|  |  | fliH | orf02497 | orf01990 |
|  |  | fliI | orf02496 | orf01991 |
|  |  | fliJ | orf02495 | orf01992 |
|  |  | fliK | orf02494 | orf01993 |
|  |  | fliL | orf02493 | orf01994 |
|  |  | fliN | orf02491 | orf01996 |
|  |  | fliO | orf02490 | orf01997 |
|  |  | fliP | orf02489 | orf01998 |
|  |  | fliQ | orf02488 | orf01999 |
|  |  | fliR | orf02487 | orf02000 |
|  |  | fliS | orf02504 | orf01983 |
|  |  | flrA | orf02503 | orf01984 |
|  |  | flrB | orf02502 | orf01985 |
|  |  | flrC | orf02501 | orf01986 |
|  |  | motA | orf03531 | orf03655 |
|  |  | motB | orf03532 | orf03654 |
|  |  | motX | orf04058 | orf04005 |
|  |  | motY | orf02379 | orf02128 |
| Enzyme | Metalloproteases | hap/vvp | - | - |
|  | Neuraminidase | nanH | - | - |
| Iron uptake | Enterobactin receptors | irgA | orf01759 | orf01596 |
|  |  | vctA | orf00535 | orf01082 |
|  | Heme receptors | hasR | - | - |
|  |  | hutA | orf00751 | orf00867 |
|  |  | hutR | orf01323 | orf00403 |
|  | Periplasmic binding protein-dependent ABC transport systems | vctC | orf00532 | orf01086 |
|  |  | vctD | orf00530 | orf01088 |
|  |  | vctG | orf00531 | orf01087 |
|  |  | vctP | orf00529 | orf01089 |
|  |  | viuC | - | - |
|  |  | viuD | - | - |
|  |  | viuG | - | - |
|  |  | viuP | - | - |
|  | Vibriobactin biosynthesis | vibA | - | - |
|  |  | vibB | - | - |
|  |  | vibC | - | - |
|  |  | vibD | - | - |
|  |  | vibE | - | - |
|  |  | vibF | - | - |
|  |  | vibH | - | - |
|  | Vibriobactin utilization | viuA | - | - |
|  |  | viuB | - | - |
| Quorum sensing | Autoinducer-2 | luxS | orf01862 | orf01681 |
|  | Cholerae autoinducer-1 | cqsA | orf00581 | orf01037 |
| Secretion system | EPS type II secretion system | epsC | orf02599 | orf03172 |
|  |  | epsE | orf02601 | orf03174 |
|  |  | epsF | orf02602 | orf03175 |
|  |  | epsG | orf02603 | orf03176 |
|  |  | epsH | orf02604 | orf03177 |
|  |  | epsI | orf02605 | orf03178 |
|  |  | epsJ | orf02606 | orf03179 |
|  |  | epsK | orf02607 | orf03180 |
|  |  | epsL | orf02608 | orf03181 |
|  |  | epsM | orf02609 | orf03182 |
|  |  | epsN | orf02610 | orf03183 |
|  |  | gspD | orf02600 | orf03173 |
|  | TTSS-1 secreted effectors | Undetermined | orf00329 | orf01280 |
|  |  | vopQ | orf03732 | orf02453 |
|  |  | vopR | orf03730 | orf02451 |
|  |  | vopS | orf03728 | orf02449 |
|  | TTSS-1 | sycN | orf03747 | orf02467 |
|  |  | tyeA | orf03746 | orf02466 |
|  |  | vcrD | orf03750 | orf02470 |
|  |  | vcrG | orf03752 | orf02472 |
|  |  | vcrH | orf03754 | orf02474 |
|  |  | vcrR | orf03751 | orf02471 |
|  |  | vcrV | orf03753 | orf02473 |
|  |  | virF | orf03714 | orf02435 |
|  |  | virG | - | - |
|  |  | vopB | orf03755 | orf02475 |
|  |  | vopD | orf03756 | orf02476 |
|  |  | vopN | orf03745 | orf02465 |
|  |  | vscA | orf03715 | orf02436 |
|  |  | vscB | orf03716 | orf02437 |
|  |  | vscC | orf03717 | orf02438 |
|  |  | vscD | orf03718 | orf02439 |
|  |  | vscF | orf03720 | orf02441 |
|  |  | vscG | orf03721 | orf02442 |
|  |  | vscH | orf03722 | orf02443 |
|  |  | vscI | orf03723 | orf02444 |
|  |  | vscJ | orf03724 | orf02445 |
|  |  | vscK | orf03725 | orf02446 |
|  |  | vscL | orf03726 | orf02447 |
|  |  | vscN | orf03744 | orf02464 |
|  |  | vscO | orf03743 | orf02463 |
|  |  | vscP | orf03742 | orf02462 |
|  |  | vscQ | orf03741 | orf02461 |
|  |  | vscR | orf03740 | orf02460 |
|  |  | vscS | orf03739 | orf02459 |
|  |  | vscT | orf03738 | orf02458 |
|  |  | vscU | orf03737 | orf02457 |
|  |  | vscX | orf03748 | orf02468 |
|  |  | vscY | orf03749 | orf02469 |
|  |  | vxsC | orf03713 | orf02434 |
|  | TTSS-2 secreted effectors | vopA | - | - |
|  |  | vopC | - | - |
|  |  | vopL | - | - |
|  |  | vopT | - | - |
|  | TTSS-2 | Undetermined | - | - |
|  |  | Undetermined | - | - |
|  |  | Undetermined | - | - |
|  |  | Undetermined | - | - |
|  |  | Undetermined | - | - |
|  |  | Undetermined | - | - |
|  |  | Undetermined | - | - |
|  |  | Undetermined | - | - |
|  |  | Undetermined | - | - |
|  |  | vcrD2 | - | - |
|  |  | vscC2 | - | - |
|  |  | vscN2 | - | - |
|  | VAS effector proteins | hcp-1 | - | - |
|  |  | hcp-2 | - | - |
|  |  | vgrG-1 | - | - |
|  |  | vgrG-2 | - | - |
|  |  | vgrG-3 | - | - |
|  | VAS type VI secretion system | vasa | - | - |
|  |  | vasB | - | - |
|  |  | vasC | - | - |
|  |  | vasD | - | - |
|  |  | vase | - | - |
|  |  | vasF | - | - |
|  |  | vasG | - | - |
|  |  | vasH | - | - |
|  |  | vasI | - | - |
|  |  | vasJ | - | - |
|  |  | vasK | - | - |
| Toxin | Accessory cholera enterotoxin | Ace | - | - |
|  | Cholera toxin | ctxA | - | - |
|  |  | ctxB | - | - |
|  | Hemolysin/cytolysin | vvhA | - | - |
|  | RTX toxin | rtxA | - | - |
|  |  | rtxB | - | - |
|  |  | rtxC | - | - |
|  |  | rtxD | - | - |
|  | Thermolabile hemolysin | Tlh | orf00118 | orf01488 |
|  | Thermostable direct hemolysin | Tdh | - | - |
|  | V.cholerae cytolysin | hlyA | - | - |
|  | Zona occludens toxin | zot | - | - |
| Immune evasion | Capsule(Acinetobacter) |  | orf02684 | - |
