# Supplementary material for: Genomic and evolutionary features of two AHPND positive Vibrio parahaemolyticus strains isolated from shrimp (Penaeus monodon) of south-west Bangladesh
Source: BMC Microbiol. 2019 Dec 3;19:270. doi: 10.1186/s12866-019-1655-8 (PMC6889531; doi:10.1186/s12866-019-1655-8)
Supplement: Supplementary file 5 — Additional file 5. Number of Genomic islands (GIs) predicted for AHPND positive V. parahaemolyticus strains MSR16 and MSR17. [file 12866_2019_1655_MOESM5_ESM.docx]

**Additional file 5: Number of Genomic islands (GIs) predicted for *V. parahaemolyticus* strains MSR16 and MSR17.**

| **Strains** | **Prediction Method** | **Chromosome I** | | **Chromosome II** | | | |
| --- | --- | --- | --- | --- | --- | --- | --- |
| **MSR16** | **SIGI-HMM** | **Island start** | **Island end** | **Island start** | | | **Island end** |
|  |  | 490922 | 499629 | 85004 | | | 103567 |
|  |  | 592462 | 627546 | 540315 | | | 544493 |
|  |  | 638244 | 645115 | 984102 | | | 988414 |
|  |  | 1231732 | 1236143 | 1131665 | | | 1136353 |
|  |  | 1375790 | 1468562 | 1146663 | | | 1161204 |
|  |  | 1469737 | 1474380 | 1162110 | | | 1167533 |
|  |  | 1632888 | 1652508 | 1189639 | | | 1207477 |
|  |  | 1716318 | 1727983 | 1209854 | | | 1223866 |
|  |  | 1746327 | 1754240 |  | | |  |
|  |  | 1853194 | 1872025 |  |  |  |  |
|  |  | 1951825 | 1962787 |  | | | |
|  |  | 1979337 | 1983349 |  | | | |
|  |  | 2749160 | 2763152 |  | | | |
|  |  | 2941181 | 2946020 |  | | | |
|  |  | 3021766 | 3058615 |  |  |  |  |
|  |  | 3185637 | 3196424 |  | | | |
|  |  | 3206753 | 3215220 |  |  |  |  |
|  |  | 3233356 | 3238277 |  | | | |
|  | **IslandPick** | 593746 | 598347 | 72563 | | | 103780 |
|  |  | 1233350 | 1238674 | 1163461 | | | 1171897 |
|  |  | 1416139 | 1422414 | 1833845 | | | 1839283 |
|  |  | 1462938 | 1467562 |  | | | |
|  |  | 1487162 | 1493448 |  | | | |
|  |  | 1496899 | 1501601 |  | | | |
|  |  | 1566656 | 1580118 |  | | | |
|  |  | 1583747 | 1588900 |  | | | |
|  |  | 1626809 | 1636488 |  | | | |
|  |  | 1652412 | 1657802 |  |  |  |  |
|  |  | 1851977 | 1861583 |  |  |  |  |
|  |  | 1866609 | 1877578 |  | | | |
|  |  | 1891623 | 1899338 |  | | | |
|  |  | 1909872 | 1926522 |  |  |  |  |
|  |  | 1938738 | 1945654 |  | | |  |
|  |  | 1947358 | 1951884 |  | | |  |
|  |  | 1958969 | 1967777 |  | | | |
|  |  | 1967898 | 1976646 |  |  |  |  |
|  |  | 1976770 | 1988213 |  | | | |
|  |  | 1988259 | 2001293 |  |  |  |  |
|  |  | 2008800 | 2013004 |  | | |  |
|  |  | 2014322 | 2034567 |  |  |  |  |
|  | **IslandPath-DIMOB** | 487696 | 496975 | 72629 | | | 105065 |
|  |  | 592915 | 627546 | 1110106 | | | 1226239 |
|  |  | 1621452 | 1671823 |  | | | |
| **MSR16** | **IslandPath-DIMOB** | 1716318 | 1730300 |  | | | |
|  |  | 1990160 | 1995458 |  |  |  |  |
|  |  | 2293101 | 2300448 |  | | | |
|  |  | 2745445 | 2760661 |  | | | |
|  |  | 3019439 | 3056612 |  | | | |
|  |  | 3231323 | 3250188 |  | | | |
| **MSR17** | **SIGI-HMM** | 24276 | 50351 | 495419 | | 499598 | |
|  |  | 167334 | 175826 | 602418 | | 606667 | |
|  |  | 299661 | 305975 | 614901 | | 619731 | |
|  |  | 324903 | 339995 | 787457 | | 798621 | |
|  |  | 353538 | 367562 | 800057 | | 805180 | |
|  |  | 491436 | 496291 | 878788 | | 886761 | |
|  |  | 507339 | 514570 | 926344 | | 941671 | |
|  |  | 590885 | 595542 | 1192168 | | 1196427 | |
|  |  | 652314 | 673547 |  | | | |
|  |  | 1248583 | 1256109 |  |  |  |  |
|  |  | 1545705 | 1559113 |  |  |  |  |
|  |  | 1568201 | 1572518 |  |  |  |  |
|  |  | 1746409 | 1756922 |  | | | |
|  |  | 1775281 | 1780235 |  | | | |
|  |  | 2003244 | 2013928 |  | | | |
|  |  | 2015412 | 2087193 |  | | | |
|  |  | 2354043 | 2368678 |  | | | |
|  |  | 2588314 | 2596537 |  | | | |
|  |  | 2600695 | 2626710 |  | | | |
|  |  | 2865958 | 2876643 |  | | | |
|  |  | 3000955 | 3005830 |  | | | |
|  |  | 3383202 | 3388168 |  | | | |
|  |  | 3395580 | 3407165 |  | | | |
|  | **IslandPick** | 1261056 | 1267434 |  | | | |
|  |  | 1514139 | 1520238 |  | | | |
|  |  | 1520575 | 1527654 |  | | | |
|  |  | 1531349 | 1536844 |  | | | |
|  |  | 1544355 | 1548931 |  | | | |
|  |  | 1549309 | 1560142 |  | | | |
|  |  | 1562859 | 1568527 |  | | | |
|  |  | 1838123 | 1852262 |  | | | |
|  |  | 1883452 | 1887596 |  | | | |
|  |  | 1893720 | 1900367 |  | | | |
|  |  | 1914060 | 1921951 |  | | | |
|  |  | 1933678 | 1940304 |  | | | |
|  |  | 1969777 | 1974613 |  | | | |
|  |  | 1981777 | 1987498 |  | | | |
|  |  | 1992662 | 2003335 |  | | | |
|  |  | 2003530 | 2017416 |  | | | |
|  |  | 2023443 | 2028429 |  | | | |
|  |  | 2061004 | 2069812 |  | | | |
|  |  | 2073871 | 2078135 |  | | | |
|  |  | 2213515 | 2218306 |  | | | |
| **MSR17** | **IslandPick** | 2220062 | 2230837 |  | | | |
|  |  | 2241938 | 2250060 |  | | | |
|  |  | 2602170 | 2607689 |  | | | |
|  |  | 2609906 | 2615900 |  | | | |
|  |  | 2878431 | 2886158 |  | | | |
|  |  | 2886847 | 2892533 |  | | | |
|  |  | 2995272 | 3004613 |  | | | |
|  |  | 3389938 | 3397249 |  | | | |
|  | **IslandPath-DIMOB** | 23133 | 50351 | 789882 | 807288 | | |
|  |  | 165535 | 175095 |  | | | |
|  |  | 285178 | 307078 |  | | | |
|  |  | 647959 | 674762 |  | | | |
|  |  | 1242451 | 1258326 |  | | | |
|  |  | 2004411 | 2068836 |  | | | |
|  |  | 2074589 | 2089302 |  | | | |
|  |  | 2360546 | 2368678 |  | | | |
|  |  | 2585166 | 2633811 |  | | | |
|  |  | 2983164 | 3005830 |  | | | |
|  |  | 3395815 | 3406294 |  | | | |
